# Evaluation of the second-generation whole-heart motion correction algorithm (SSF2) used to demonstrate the aortic annulus on cardiac CT

**DOI:** 10.1038/s41598-023-30786-7

**Published:** 2023-03-03

**Authors:** Yoriaki Matsumoto, Chikako Fujioka, Kazushi Yokomachi, Nobuo Kitera, Eiji Nishimaru, Masao Kiguchi, Toru Higaki, Ikuo Kawashita, Fuminari Tatsugami, Yuko Nakamura, Kazuo Awai

**Affiliations:** 1 Department of Radiology, Hiroshima University Hospital, 1-2-3 Kasumi, Minami-ku, Hiroshima, Japan; 2 Department of Diagnostic Radiology, Graduate School of Biomedical and Health Sciences, Hiroshima University, 1-2-3 Kasumi, Minami-ku, Hiroshima, Japan

**Keywords:** Cardiac device therapy, Interventional cardiology, Medical imaging, Three-dimensional imaging, Tomography, Clinical trial design, Cardiovascular diseases

## Abstract

The main purpose of pre-transcatheter aortic valve implantation (TAVI) cardiac computed tomography (CT) for patients with severe aortic stenosis is aortic annulus measurements. However, motion artifacts present a technical challenge because they can reduce the measurement accuracy of the aortic annulus. Therefore, we applied the recently developed second-generation whole-heart motion correction algorithm (SnapShot Freeze 2.0, SSF2) to pre-TAVI cardiac CT and investigated its clinical utility by stratified analysis of the patient's heart rate during scanning. We found that SSF2 reconstruction significantly reduced aortic annulus motion artifacts and improved the image quality and measurement accuracy compared to standard reconstruction, especially in patients with high heart rate or a 40% R-R interval (systolic phase). SSF2 may contribute to improving the measurement accuracy of the aortic annulus.

## Introduction

Electrocardiogram-gated cardiac computed tomography (CT) scans are important for planning the transcatheter aortic valve implantation (TAVI) procedure in patients with severe aortic stenosis^1,2^. However, motion artifacts present a technical challenge because they can compromise the assessment of structures such as the coronary arteries and valves, especially in patients with a high heart rate^3–7^. Inaccurate sizing increases the risk of complications such as perivalvular leak or rupture in TAVI patients^2,8,9^. Precise pre-procedural imaging is therefore crucial to assure optimal patient outcome^2,9^. To avoid motion artifacts, the society of cardiovascular CT guidelines^10^ recommend that the heart rate be controlled to be less than 60 beats per minute (bpm) by the oral or intravenous administration of a β-blocker. To correct motion artifacts, technical advances in CT systems have improved the temporal resolution, increased the gantry rotation speed, and applied dual-source CT and multi-segment reconstruction; software solutions have been developed^11^. A recent study reported that a generative adversarial network model could create images with fewer motion artifacts while preserving the lesion contrast^12^. Although such an approach using machine learning may effectively reduce cardiac motion artifacts, it has not yet been implemented in clinical practice.

The first-generation motion correction algorithm (SnapShot Freeze, SSF1; GE Healthcare) is vendor-specific and designed to address coronary motion artifacts on cardiac scans. Its application significantly improved the image quality of the coronary arteries in patients with a high heart rate^13–20^. However, SSF1 cannot address other non-coronary intracardiac structures. The second-generation motion correction algorithm (SnapShot Freeze 2.0, SSF2; GE Healthcare) increased the motion-correction range to include the whole heart within one scan volume^21,22^.

To our knowledge, the clinical effectiveness of SSF2 with respect to the aortic annulus has not been investigated. We hypothesized that use of the SSF2 algorithm would improve the image quality of CT scans acquired to evaluate aortic valves, because a reduction in motion artifacts can improve the CT image quality and diagnostic accuracy. In this study we enrolled patients with severe aortic stenosis and compared the quality of standard images without motion correction with pre-TAVI cardiac CT scans subjected to SSF2.

## Materials and methods

This retrospective study conformed to the principles of the Declaration of Helsinki. In accordance with the Ethical Guidelines for Medical and Health Research Involving Human Subjects (Ministry of Education, Culture, Sports, Science and Technology and Ministry of Health, Labour and Welfare, Japan), study information including the objectives was disclosed on our hospital website with an opt-out approach. The Ethical Committee for Epidemiology of Hiroshima University reviewed and approved the study protocol (No. E-2623, Clinical study of motion correction algorithm for cardiac CT). Informed patient consent for the analyses was waived.

### Study population

We enrolled 108 patients with severe aortic stenosis who underwent cardiac CT as candidates for TAVI between April 2021 and February 2022. Inclusion criteria were patients who underwent contrast-enhanced cardiac CT. Our exclusion criteria were severe renal failure (estimated glomerular filtration rate < 30 ml/min/1.73 m^2^, 15 patients), poor breath holding during scanning (1 patient), extravasation during contrast injection (1 patient) or refusal of CT examination (1 patient). Thus, the final study population consisted of 90 patients; they were 33 male and 57 female ranging in age from 70 to 95 years (median age, 84 years).

To perform stratified analysis of the effect of SSF2 on heart rate during scanning, we divided 90 patients into 3 groups to include the same number of patients according to the relationship between heart rate and image quality^10,13,18,19,21,23–25^. In group 1 (n = 30) the heart rate was low (< 60 bpm, range 34–59 bpm), in group 2 (n = 30) it was intermediate (60–69 bpm), and in group 3 (n = 30) it was high (70 bpm or higher, range 70–119 bpm).

### CT scanning

All patients were scanned on a 256-detector row CT scanner (Revolution CT; GE Healthcare, Milwaukee, WI, USA); prospective electrocardiogram-gated axial scans were acquired. As shown in Table 1, the scanning- and reconstruction parameters were tube voltage, 120 kVp; tube current, selected by automatic tube current modulation (Smart-mA, GE Healthcare) based on the scout image; noise index, 25; detector collimation, 256 × 0.625 mm or 224 × 0.625 mm depending on the patient’s heart size; gantry rotation, 0.28 s; slice thickness, 0.625 mm; scan field of view, 360 mm; display field of view, 200 mm; matrix, 512 × 512; reconstruction, half; reconstruction kernel, HD standard; reconstruction method, deep learning image reconstruction (TrueFidelity, strength High; GE Healthcare)^26–29^. The padding range was 0–100% of the R-R interval when a heart rate of less than 60 bpm was recorded during pre-examination monitoring; when it exceeded 60 bpm or was variable. In the presence of arrhythmia the padding range was 0–250%. All scans were craniocaudal from the tracheal bifurcation to the level of the inferior margin of the cardiac apex. All patients were able to perform breath-holds during the examination. To achieve high image quality with minimal radiation doses, patients with a heart rate above 60 bpm 5 min before the start of scanning were given 2–10 mg propranolol hydrochloride (Inderal; Taiyo Holdings Co., Ltd., Tokyo, Japan).

**Table 1:** CT acquisition parameters.

Tube voltage	120 kVp
Tube current	Automatic tube current modulation, noise index 25
Detector collimation	256 × 0.625 mm or 224 × 0.625 mm
Gantry rotation	0.28 s
Slice thickness	0.625 mm
Scan field of view	360 mm
Display field of view	200 mm
Matrix	512 × 512
Reconstruction	Half
Reconstruction kernel	HD standard
Reconstruction method	Deep learning reconstruction (Truefidelity, strength high)

The contrast medium (iodine concentration 350 mg/ml; Iomeron-350; Eisai Co., Ltd., Tokyo, Japan) was injected in triple-phase through a 20- or 22-gauge catheter into the antecubital vein using a power injector (Dual Shot type GX; Nemoto Kyorindo, Tokyo, Japan). The iodine dose of 273 mg/kg in the first phase was administered in 13 s. This injection was followed at a speed of 5 s by a 50/50 mix of contrast medium (53 mgI/kg) and saline, and finally 100% saline was delivered at the same injection speed. For cardiac CT scanning, bolus-tracking was used to synchronize the arrival of contrast medium at the left atrium and left ventricle with the start of scanning. To monitor arrival, we acquired axial scans at one-second intervals at the level of the left atrium and the left ventricle 10 s after the start of contrast medium injection. The radiation dose was 120 kVp, 40 mA. Scanning was started automatically 5 s after contrast enhancement reached 150 Hounsfield units in a region of interest (ROI) placed in the center of the left atrium and the left ventricle.

### Data processing

Similar to the SSF1 algorithm^18,20^, the SSF2 algorithm uses data from adjacent cardiac phases (64 ms before and after the target phase) to characterize and correct the motion. The SSF2 algorithm, a fully automated technique based on information and feedback obtained from SSF1 scans, seeks each region at all image volumes for a local path that is consistent with the subset of measured data. Once the vessel’s motion path is identified, the data are discretized into a series of datasets based on when the corresponding projection rays were measured. Each volume dataset in the series undergoes spatial deformation by the motion field. This allows the motion state to be mapped from the respective time to the central reference time, which is determined by the prescribed cardiac phase^30^.

All images were reconstructed using the standard (without motion correction) algorithm with deep-learning image reconstruction for reducing the image noise^26–29^. For the cardiac phase, the systolic- (R-R interval, 40%) and diastolic phase (R-R interval, 75%) used for pre-TAVI cardiac CT measurements were selected^2,4,30,31^. As the systolic- and diastolic phases were additionally subjected to SSF2 reconstruction, 4 datasets were obtained for each patient. They were anonymized and transferred to the workstation (Advantage Workstation 4.7, GE Healthcare) for later analysis.

### Quantitative evaluation

The attenuation effect elicited by motion artifacts was analyzed at the aortic annulus. All images were inspected by one radiological technologist (Y.M. with 15 years of experience with cardiac CT studies). To assess the aortic annulus, only axial- and 2D double-oblique multiplanar reconstruction (MPR) images were examined. The aortic annulus was defined as a virtual ring formed by joining the basal attachments of the aortic leaflets^2,32^.

### Edge rise distance

**Figure 1:**
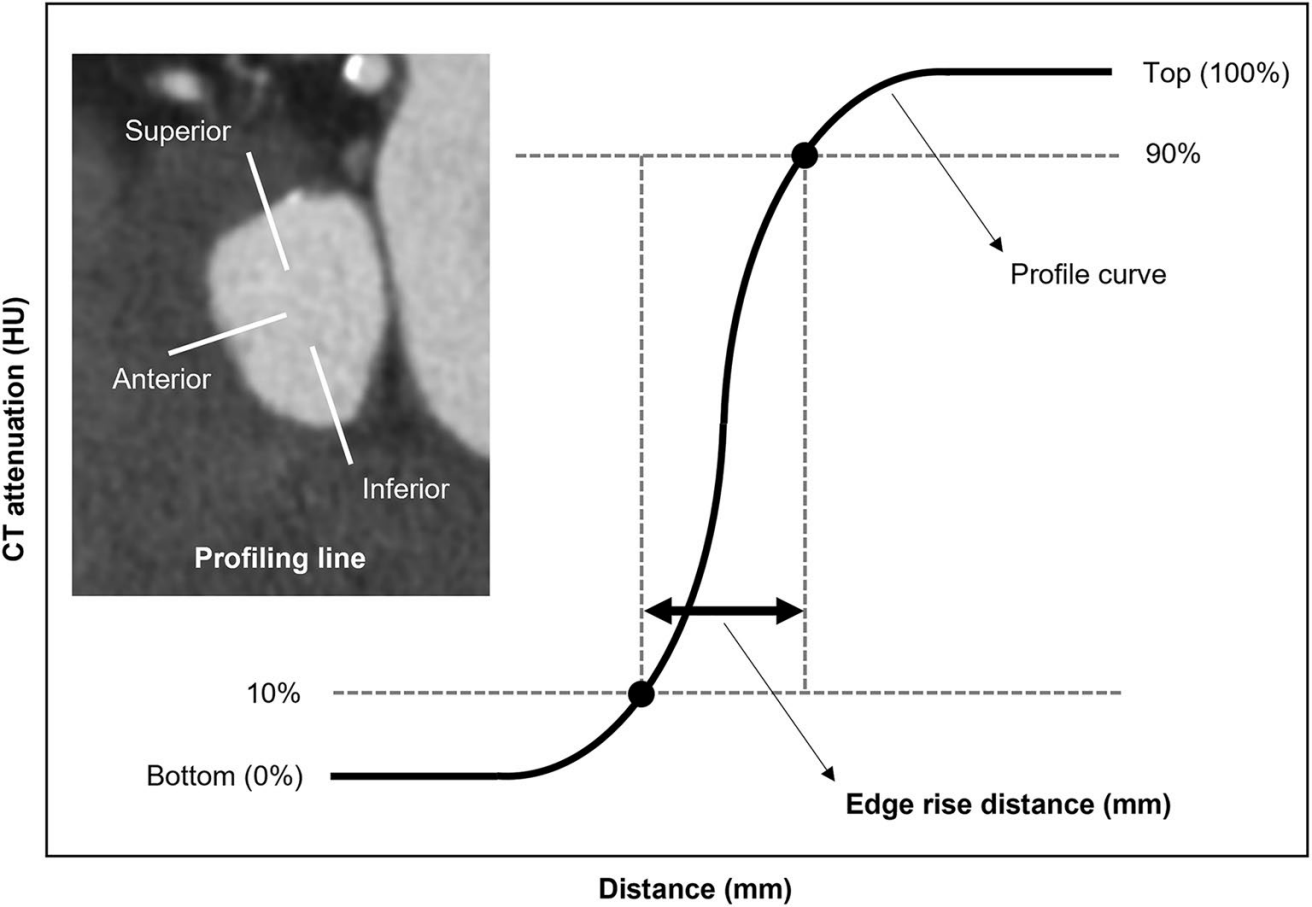
Sample image of ERD. Profile curve of the aortic annulus. The ERD at a pixel attenuation from 10 to 90% of the maximum CT attenuation is shown. CT = computed tomography; HU = Hounsfield units; ERD = edge rise distance.

We generated a 3-directional CT attenuation profile (anterior-, superior-, and inferior direction) of the aortic annulus (Fig. 1) using the particle analysis tool (Plot Profile) on the workstation (Ziostation2, Ziosoft, Tokyo, Japan). Areas of calcification where CT attenuation fluctuates significantly were carefully avoided. The CT attenuation profiles were generated at precisely the same location for images reconstructed with standard and SSF2. We cut off the bottom and top 10% of the profile and measured the 10–90% edge rise distance (ERD)^33,34^. The ERD was examined in three directions of the aortic annulus and the mean values were compared on standard- and SFF2 images.

### Dispersion of sizing

With respect to the sizing of the aortic annulus, we evaluated the dispersion between the two reconstructions. All images were analyzed by two radiological technologists (Y.M. and C.F., with 15 and 18 years of experience in cardiac CT imaging, respectively). They were blinded to presence of SSF2 technique and manually measured the aortic annulus area and perimeter of all patients independently on a CT workstation (Ziostation2, Ziosoft, Tokyo, Japan). The annulus area and perimeter were determined from the trace by placing plots (dots) at the blood-tissue interface^1,9^. The annulus border was traced outside calcifications. When boundaries were unclear due to motion artifacts the observers traced subjectively. After the plots and annular borderline were drawn manually, the annulus area and perimeter were automatically calculated on the workstation^1,9^.

### Contrast-to-noise ratio

To investigate the potential effect of SSF2 reconstruction on the quantitative ERD measurements, we inspected axial images and recorded the CT number and image noise [standard deviation (SD) of the CT number] in a circular ROI placed in the ascending aorta and septal wall of the ventricle. The size of the circular ROI cursor was as large as allowed by the diameter of the ascending aorta (approximately 5.0–10.0 mm^2^) and of the septal wall of the ventricle (approximately 1.5–3.0 mm^2^). Based on the obtained values we also calculated the contrast-to-noise ratio (CNR) using the formula: (CT number of the ascending aorta minus the CT number of the septal wall of the ventricle) divided by the image noise of the ascending aorta^35^.

### Qualitative analysis

Two radiological technologists (Y.M. and C.F., with 15 and 18 years of experience in cardiac CT imaging, respectively) were blinded to presence of the SSF2 technique. They subjectively and independently inspected the MPR images from the sinotubular junction to the left ventricular outflow tract of the datasets for motion artifacts at the aortic annulus level. To grade the image quality they used the 5-point Likert scale where 1 = very poor (motion artifacts resulting in poor visualization of the aortic valve anatomy, not evaluable), 2 = poor (degraded visualization of the aortic valve anatomy due to motion artifacts, not evaluable), 3 = fair (minor motion artifacts with clear delineation of the aortic valve anatomy), 4 = good (no motion artifacts with confident identification of the aortic root anatomy including the cusp nadirs and annular contours), and 5 = excellent (outstanding image quality with a high level of diagnostic certainty with regard to the aortic valve cusps, the leaflet nadirs, and the detection of the aortic annular contours)^30^. Interobserver disagreement was resolved by consensus.

### Statistical analysis

Continuous variables of demographic data, ERD, CT number, image noise and CNR are expressed as the median and range or as percentages or counts, aortic annulus area and perimeter or image quality scores as the mean and SD. The results of ERD, CT number, image noise, CNR and image quality scores were compared on images reconstructed with standard and SSF2 using the Mann–Whitney *U*-test. To compare the dispersion (SD) of area and perimeter between the two reconstructions we used the *F*-test. Intra- and interobserver agreement on the variability of the quantitative evaluation by Bland–Altman plot analysis was expected to converge to a 95% limit of agreement, defined as a mean difference of ± 1.96 SD. To determine whether the CNR was equivalent in standard and SSF2 reconstructions, we performed the equivalence test^36^. As the SD of the CNR between the proximal coronary arteries and the adjacent perivascular tissue was 5 in our earlier study^34^, we adopted 5 as the equivalent margin. Interobserver agreement in the qualitative evaluation was classified as evaluable (score 3–5) and non-evaluable (score 1, 2) assessed with the Cohen kappa κ coefficient where a κ value of less than 0.20 = poor, 0.21–0.40 = fair, 0.41–0.60 = moderate, 0.61–0.80 = substantial, and 0.81–1.00 = near perfect agreement. All statistical analyses were performed with JMP 16 (SAS Institute Inc., Cary, NC, USA). Differences of *p* < 0.05 were considered statistically significant.

## Results

### Patient demographic data

As shown in Table 2, the median overall heart rate during CT image acquisition was 64 bpm (range: 34–119 bpm). Of the 90 patients, 70 were in sinus rhythm and 20 exhibited arrhythmias (atrial fibrillation, 19 patients; premature atrial contraction, 1 patient).

**Table 2:** Patient characteristics.

	Overall	Patients with low HR (< 60 bpm)	Patients with intermediate HR (60–69 bpm)	Patients with high HR (> 70 bpm)
Number of patients	90	30	30	30
Age (years)	84 (70–95)	85 (70–95)	84 (70–91)	84 (74–94)
Male, n (%)	33 (37%)	14 (42%)	11 (33%)	8 (25%)
Height (cm)	151 (130–169)	153 (137–167)	149 (130–169)	150 (135–169)
Body weight (kg)	51 (33–76)	50 (33–68)	53 (33–73)	52 (35–76)
Body mass index (kg/m^2^)	22.4 (13.6–36.9)	21.7 (13.6–26.6)	22.9 (15.5–36.9)	22.7 (15.8–34.6)
estimated glomerular filtration rate (ml/min/1.73 m^2^)	51.0 (30.3–115.5)	46.9 (30.3–115.5)	50.6 (31.3–87.3)	52.9 (31.0–82.8)
Heart rate during the scan (beats/min)	64 (34–119)	53 (34–59)	64 (60–69)	80 (70–119)
No. of patients with arrhythmias during the scan, n (%)	20 (22%)	6 (30%)	3 (15%)	11 (55%)
Atrial fibrillation, n (%)	19 (21%)	6 (31%)	3 (16%)	10 (53%)
Premature atrial contraction, n (%)	1 (1%)	0 (0%)	0 (0%)	1 (100%)
No. of patients using propranolol hydrochloride, n (%)	69 (77%)	17 (25%)	23 (33%)	29 (42%)

Values are the median (range) or the number of patients (%).

Propranolol hydrochloride was administered to reduce the heart rate before imaging.

### Quantitative evaluation

#### Edge rise distance

**Figure 2:**
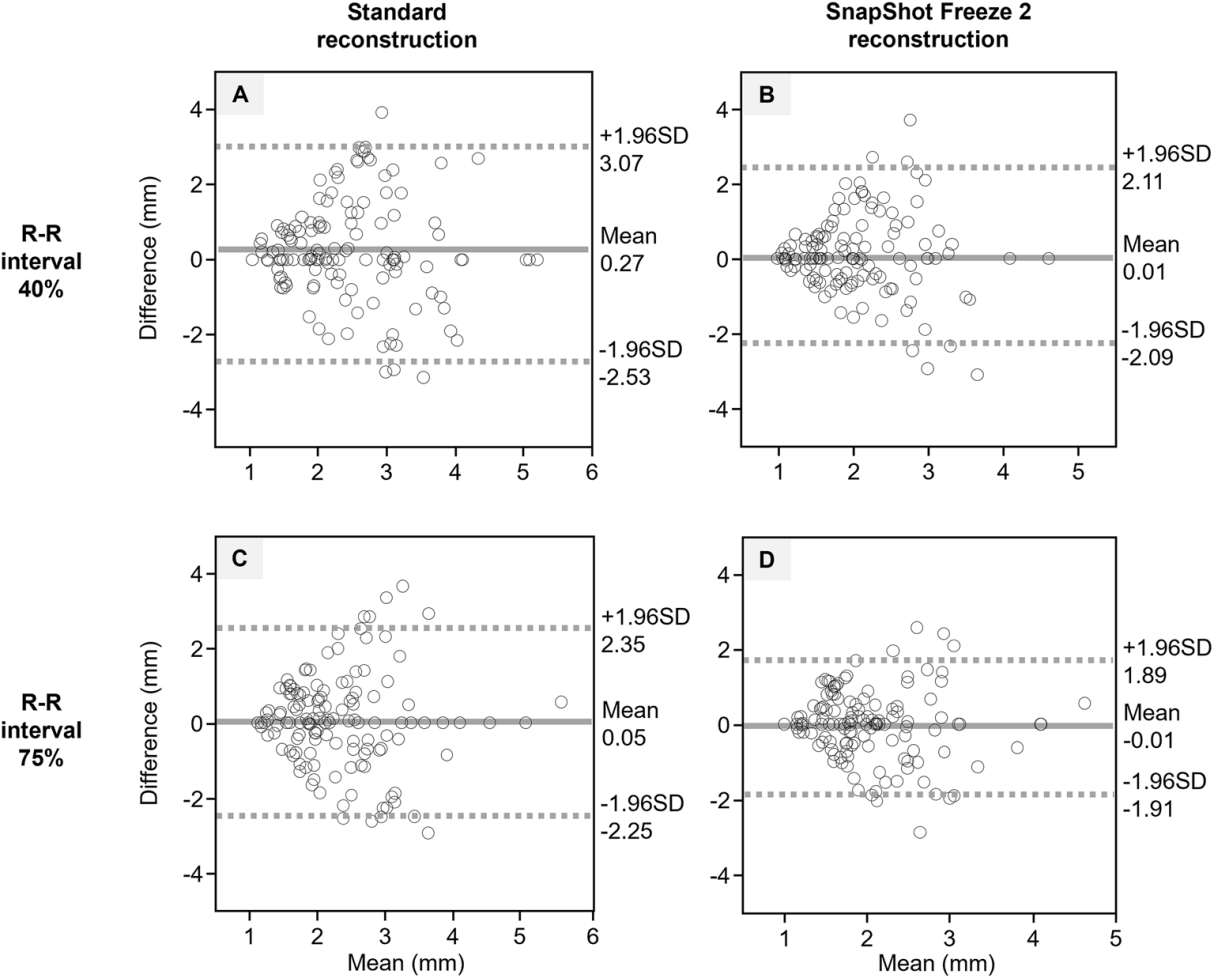
Bland–Altman plot analysis of the intraobserver agreement with respect to the edge rise distance. Standard- and SSF2 reconstruction at an R-R interval of 40% (**A** and **B**) and standard- and SSF2 reconstruction at an R-R interval of 75% (**C** and **D**). The solid line represents the mean difference, the dashed lines represent the 95% limit of agreement (mean difference ± 1.96 SD). SD = standard deviation.

We analyzed 1080 ERDs (3 directions × 4 datasets × 90 patients). The ERD measurement results are presented in Table 3. In patients with a low heart rate, the ERD obtained with standard and SSF2 reconstruction was not significantly different (R-R 40% and R-R 75%: *p* > 0.05). However, in patients with an intermediate heart rate, the ERD at R-R 40% was significantly shorter on SSF2 (2.0 mm)- than standard (2.4 mm) images (*p* < 0.001). In patients whose heart rate was high, the ERD at R-R 40% and R-R 75% was significantly shorter on SSF2- than standard images (*p* < 0.001). Bland–Altman plots for intraobserver agreement with respect to the ERD for 4 datasets are summarized in Fig. 2. The plots nearly converged within the 95% limit of agreement for all datasets.

**Table 3:** Comparison of the edge rise distance (mm) on scans subjected to standard- and SSF2 reconstruction.

	R-R interval (%)	Standard	SSF2	*P*
Patients with low HR (< 60 bpm)	40	1.8 (1.0–4.2)	1.6 (0.9–4.0)	0.067
	75	1.9 (1.0–4.2)	1.8 (0.9–4.1)	0.122
Patients with intermediate HR (60–69 bpm)	40	2.4 (0.9–5.1)	2.0 (0.9–4.5)	< 0.001
	75	2.1 (1.0–5.1)	2.1 (0.9–4.1)	0.077
Patients with high HR (> 70 bpm)	40	2.5 (1.1–5.7)	2.0 (1.0–5.2)	< 0.001
	75	2.5 (1.1–5.9)	2.0 (1.0–4.9)	< 0.001

*HR* Heart rate. Values are the median (range).

#### Dispersion of sizing

**Figure 3:**
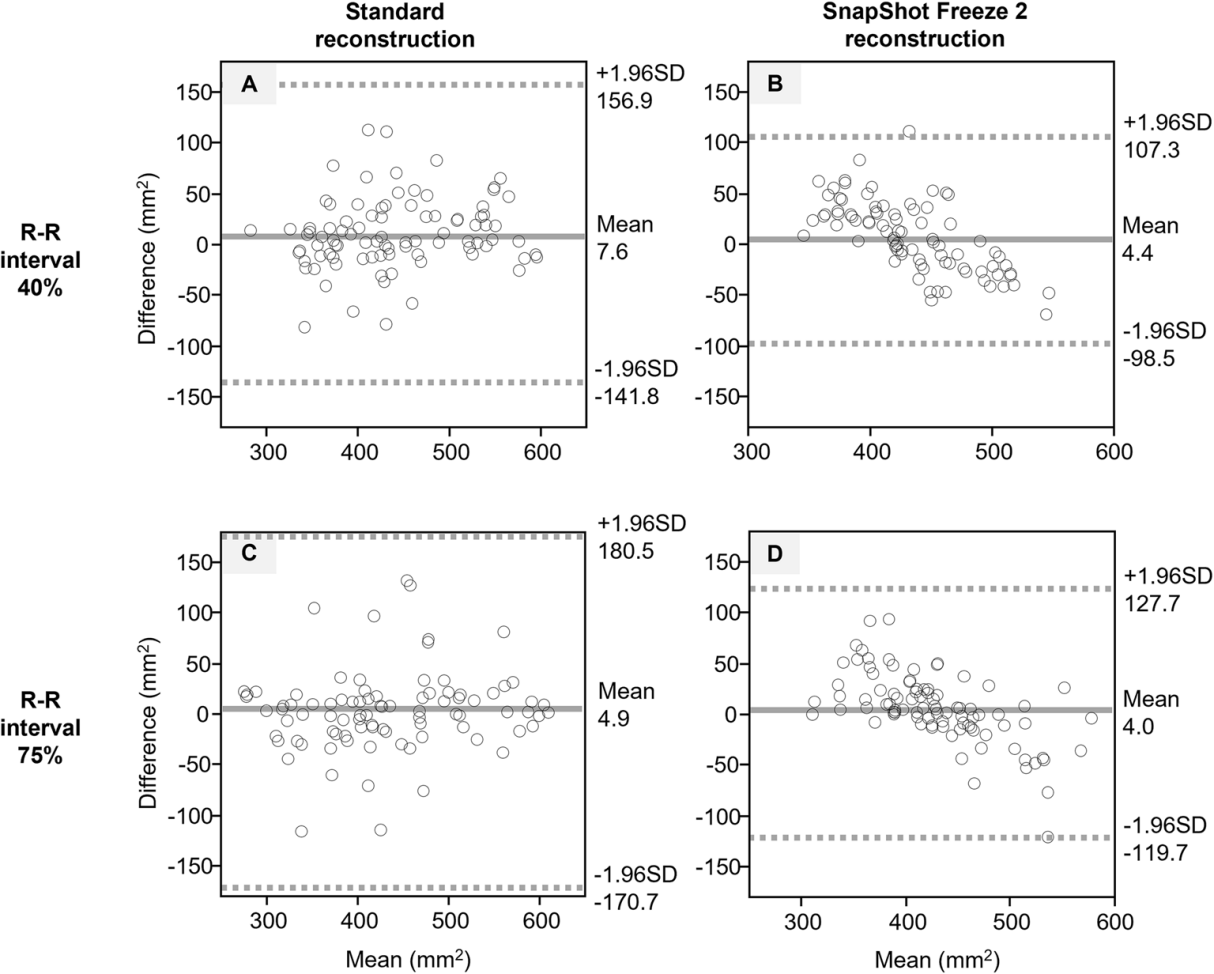
Bland–Altman plot analysis of the interobserver agreement with respect to the aortic annulus areas. Standard- and SSF2 reconstruction at an R-R interval of 40% (**A** and **B**) and standard- and SSF2 reconstruction at an R-R interval of 75% (**C** and **D**). The solid line represents the mean difference, the dashed lines represent the 95% limit of agreement (mean difference ± 1.96 SD). SD = standard deviation.

**Figure 4:**
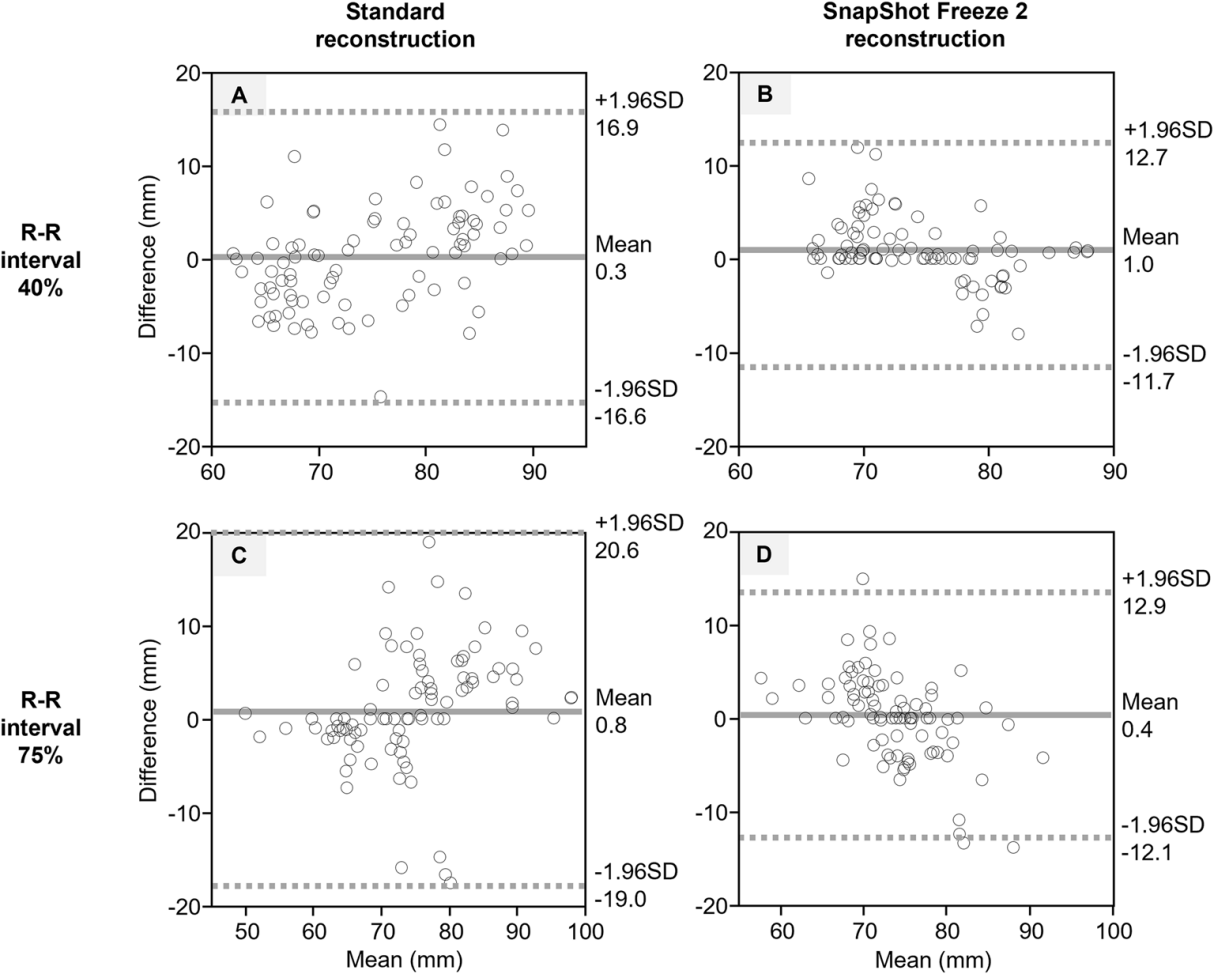
Bland–Altman plot analysis of interobserver agreement with respect to the aortic annulus perimeter. Standard- and SSF2 reconstruction at an R-R interval of 40% (**A** and **B**) and standard- and SSF2 reconstruction at an R-R interval of 75% (**C** and **D**). The solid line represents the mean difference and the dashed lines the 95% limit of agreement (mean difference ± 1.96 SD). SD = standard deviation.

As shown in Table 4, the SD of the aortic annulus area was significantly smaller in SSF2 reconstruction than in standard at low (94.7 vs. 63.3 and 105.2 vs. 78.9)-, intermediate (71.8 vs. 47.9 and 90.4 vs. 58.3)-, and high heart rate (58.7 vs. 45.1 and 70.3 vs. 45.8) R-R interval of 40 and 75% (all: *p* < 0.05). As shown in Table 5, the SD of the aortic annulus perimeter was also significantly smaller in SSF2 reconstruction than in standard at low (11.6 vs. 7.4 and 9.5 vs. 6.0)-, intermediate (9.4 vs. 5.6 and 10.8 vs. 6.8)-, and high heart rate (8.4 vs. 4.3 and 9.3 vs. 5.4) R-R interval of 40 and 75% (all: *p* < 0.001). Bland–Altman plots for interobserver agreement with respect to the annular area and perimeter are shown in Figs. 3 and 4. For the area and perimeter, the plots almost converged within the 95% limit of agreement for all datasets. In particular, the mean difference (± 1.96 SD) was within a smaller range on SSF2- than standard reconstruction images.

**Table 4:** Comparison of SD of the aortic annulus areas (mm^2^) of scans subjected to standard- and SSF2 reconstruction.

	R-R interval (%)	Standard	SSF2	*P*
Patients with low HR (< 60 bpm)	40	448.7 (94.7)	436.5 (63.3)	0.002
	75	428.9 (105.2)	435.0 (78.9)	0.029
Patients with intermediate HR (60–69 bpm)	40	442.1 (71.8)	435.5 (47.9)	0.002
	75	445.8 (90.4)	439.4 (58.3)	0.001
Patients with high HR (> 70 bpm)	40	437.4 (58.7)	435.8 (45.1)	0.002
	75	432.3 (70.3)	414.6 (45.8)	0.001

*HR* Heart rate, *bpm* Beats per minute. Values are the mean (SD).

**Table 5:** Comparison of SD of the aortic annulus perimeter (mm) of scans subjected to standard- and SSF2 reconstruction.

	R-R interval (%)	Standard	SSF2	*P*
Patients with low HR (< 60 bpm)	40	75.0 (11.6)	75.6 (7.4)	< 0.001
	75	75.4 (9.5)	74.7 (6.0)	< 0.001
Patients with intermediate HR (60–69 bpm)	40	74.6 (9.4)	74.0 (5.6)	< 0.001
	75	77.0 (10.8)	75.7 (6.8)	< 0.001
Patients with high HR (> 70 bpm)	40	74.8 (8.4)	73.3 (4.3)	< 0.001
	75	71.5 (9.3)	71.3 (5.4)	< 0.001

*HR* Heart rate, *bpm* Beats per minute. Values are the mean (SD).

#### Contrast-to-noise ratio

**Figure 5:**
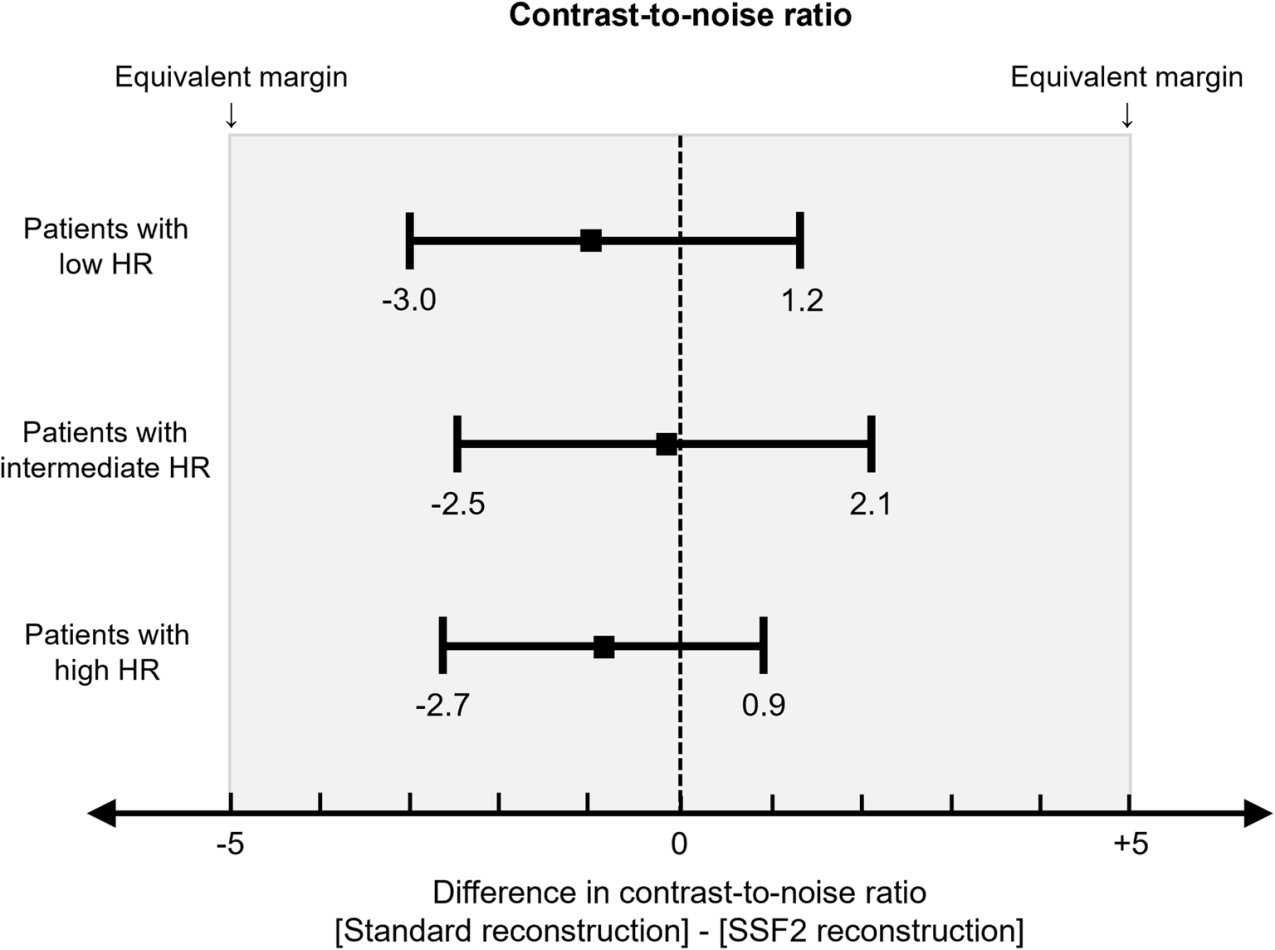
Results of the equivalence test. Results of the equivalence test for the difference in CNR between standard and SSF2 reconstruction. CNR = contrast-to-noise ratio; HR = heart rate; SSF2 = SnapShot Freeze 2.

As shown in Table 6, the CT number of the ascending aorta and the septal wall of the ventricle and the image noise of the ascending aorta showed no significant difference between the two reconstructions, irrespective of the patients’ heart rate (all: *p* > 0.05). In addition, these CNR also showed no significant difference between the two reconstructions at low (18.5 vs. 19.5, *p* = 0.404)-, intermediate (16.5 vs. 16.3, *p* = 0.860)-, and high heart rate (17.6 vs. 18.1, *p* = 0.312). The 95% confidence interval for the difference between standard and SSF2 reconstruction was − 3.0 to 1.2 in patients with a low heart rate, − 2.5 to 2.1 in patients with an intermediate heart rate, and − 2.7 to 0.9 in patients with a high heart rate. Because the 95% confidence interval did not cross the bilateral predefined equivalence margin (Fig. 5) in all heart rate classes, we considered CNR to be equivalent among our standard and SSF2 reconstitution irrespective of their heart rate.

**Table 6:** CT number, image noise and contrast-to-noise ratio at each site.

		Standard	SSF2	*P*
Patients with low HR (< 60 bpm)	CT number of the ascending aorta (HU)	401.7 (204.7–478.9)	400.7 (205.0–478.9)	0.928
	Image noise of the ascending aorta	16.9 (13.1–22.3)	16.3 (11.6–22.2)	0.206
	CT number of septal wall of the ventricle (HU)	83.4 (59.5–116.0)	85.6 (56.7–115.0)	0.601
	Contrast-to-noise ratio	18.5 (8.5–24.1)	19.5 (9.0–26.9)	0.404
Patients with intermediate HR (60–69 bpm)	CT number of the ascending aorta (HU)	375.0 (308.8–490.0)	380.6 (299.0–492.5)	0.962
	Image noise of the ascending aorta	17.3 (13.0–26.5)	17.7 (13.0–27.5)	0.818
	CT number of septal wall of the ventricle (HU)	81.1 (55.9–111.1)	82.5 (49.3–115.0)	0.904
	Contrast-to-noise ratio	16.5 (11.5–30.8)	16.3 (9.9–31.0)	0.860
Patients with high HR (> 70 bpm)	CT number of the ascending aorta (HU)	400.0 (314.5–531.7)	400.0 (295.5–528.7)	0.885
	Image noise of the ascending aorta	18.1 (15.5–23.5)	17.0 (13.9–22.0)	0.161
	CT number of septal wall of the ventricle (HU)	80.5 (59.5–117.4)	83.4 (56.7–123.2)	0.982
	Contrast-to-noise ratio	17.6 (11.7–24.0)	18.1 (13.3–24.0)	0.312

*HR* Heart rate, *HU* Hounsfield units. Values are the median (range).

### Qualitative analysis

**Figure 6:**
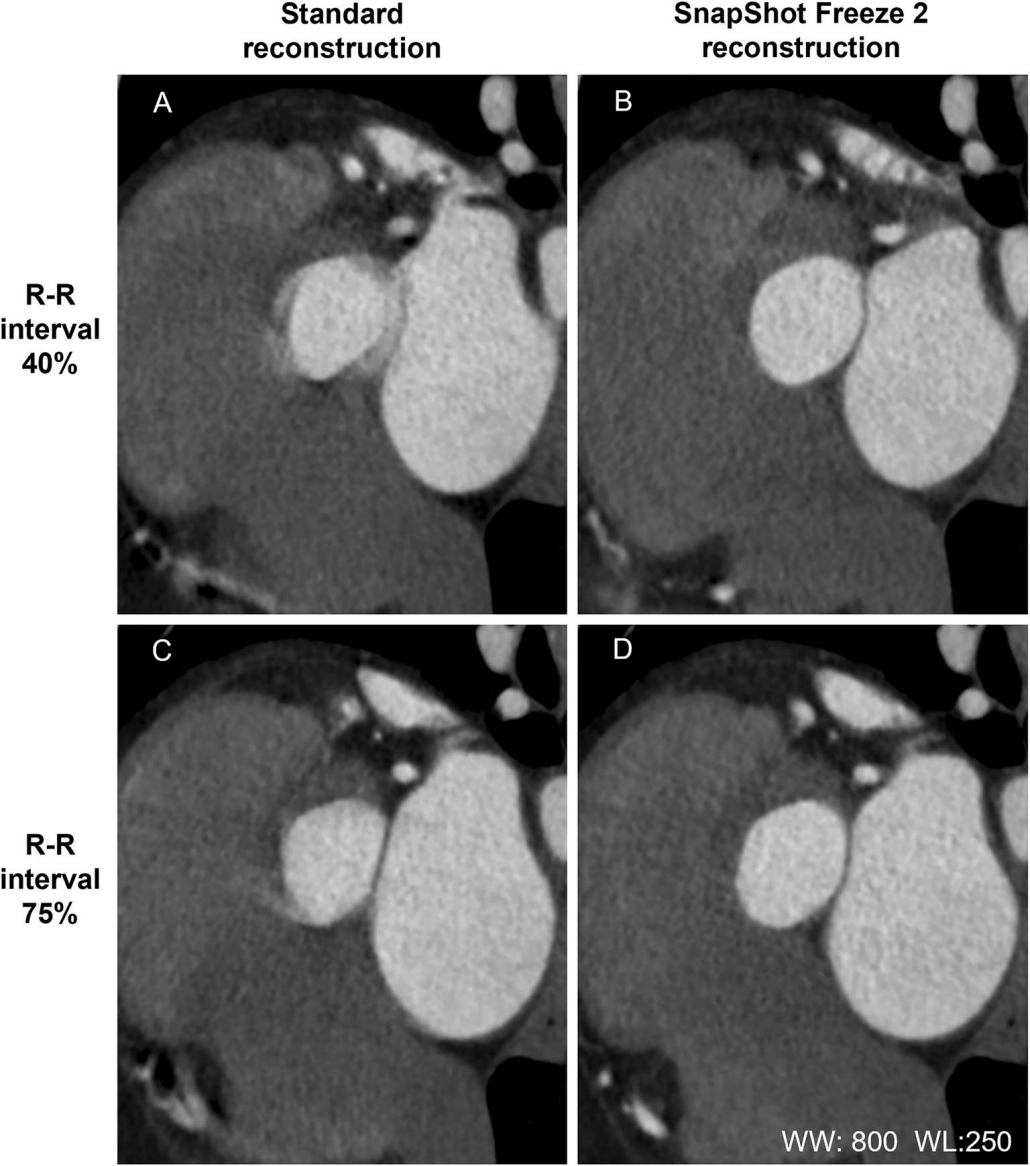
Clinical image of SSF2. In their 80 s (height = 157 cm, body weight = 58 kg, body mass index = 23.5 kg/m^2^), heart rate during the scan = 116 bpm (atrial fibrillation). (**A**) and (**C**): MPR images of the aortic annulus (R-R interval = 40% and 75%) using standard reconstruction. The visualization scores for (**A**) and (**C**) were 1 and 2, respectively. (**B**) and (**D**): After SSF2 reconstruction, both visualization scores were 4. The evaluable image quality improved. WW = window width; WL = window level.

Table 7 shows the results of the visual evaluation of MPR images submitted by our two readers. In patients with a low heart rate, at R-R 75%, there was no significant difference in the mean image scores assigned to images subjected to standard- or SSF2 reconstruction (*p* = 0.540). At R-R 40% the visualization scores were significantly higher for images reconstructed with SSF2 than standard (all: *p* < 0.01). There was substantial interobserver agreement with respect to the overall image quality (κ = 0.69). SSF2 reconstruction improved the image quality of the aortic annulus in the representative case shown in Fig. 6. All results are provided in the Supplementary file.

**Table 7:** Comparison of the image quality scores of scans subjected to standard- and SSF2 reconstruction.

	R-R interval (%)	Standard	SSF2	*P*
Patients with low HR (< 60 bpm)	40	2.6 (1.1)	3.6 (0.7)	< 0.001
	75	3.9 (0.9)	4.0 (0.7)	0.540
Patients with intermediate HR (60–69 bpm)	40	2.1 (0.9)	3.5 (0.6)	< 0.001
	75	2.9 (0.8)	3.6 (0.7)	0.003
Patients with high HR (> 70 bpm)	40	2.5 (0.7)	3.7 (0.4)	< 0.001
	75	2.2 (0.7)	3.2 (0.6)	< 0.001

*HR* Heart rate, *bpm* Beats per minute. Values are the mean (SD).

## Discussion

Our study demonstrates that the second-generation whole-heart motion correction algorithm (SSF2) was superior to standard reconstruction with respect to the image quality of pre-TAVI cardiac CT scans acquired for the evaluation of the aortic annulus.

At R-R 40%, SSF2 reconstructed images received significantly higher image quality scores than did standard reconstruction regardless of the patients’ heart rate (*p* < 0.001). At R-R 75%, in patients with an intermediate and high heart rate was the visualization score higher for SSF2- than standard reconstructed images. At R-R 40% and R-R 75%, SSF2 strongly tended to yield higher image quality scores than did standard reconstruction. Consequently, SSF2 reconstruction raised the image quality significantly, especially in patients with a high heart rate or a 40% R-R interval.

The earlier vendor-specific motion correction algorithm (SSF1) was designed to address coronary motion artifacts on cardiac scans. It was primarily indicated for coronary imaging and was shown to improve the image quality and diagnostic accuracy of scans performed for the detection of significant coronary stenosis, especially in patients with a high heart rate^13–20^. The SSF2 algorithm extends motion correction to include the whole heart. It is expected to be useful for imaging of not only the coronary arteries but also of other non-coronary intracardiac structures such as the cardiac valves.

Earlier studies that applied SSF2 reconstruction to images of the coronary arteries, of heart- and valve structures, and of the great vessels showed that the image quality was significantly improved by the algorithm and the number of non-evaluable scans was lower than of images subjected to standard- or SSF1 reconstruction^21,22^. Our study focused on the aortic annulus; it indicates that SSF2 yielded higher motion artifact correction in the whole heart.

Others^30^ who applied SSF1 to cardiac CT for aortic annulus measurements reported that it significantly improved the image quality of systolic CT datasets. We examined the effect of SSF2 in a wide range of heart rates and showed that it is useful for the evaluation of the aortic annulus not only in the systolic- but also in the diastolic phase. Our findings suggest that SSF2 reconstruction reduces aortic valve motion artifacts throughout the cardiac phases.

SSF2 reconstruction was not useful at R-R interval 75% in patients with a low or intermediate heart rate. At those heart rates and cardiac phases, the temporal resolution on electrocardiogram-gated scans may be sufficient and motion artifacts may not be inherent. SSF1- and SSF2 reconstruction may be useful in patients with a high heart rate and for scans with low temporal resolution^13,14,21,22^. Our findings suggest that SSF2 is as useful as SSF1 in patients with a high heart rate.

SSF1 cannot address other non-coronary intracardiac structures. It calculates the movement trajectories of coronary arteries using the imaging information of the target phase and the bilateral neighboring phases to reduce motion artifacts by compensating for cardiac motion and to generate diagnosable CT images of the coronary arteries^13–20^. On the other hand, the SSF2 algorithm, a fully automated technique based on knowledge and feedback obtained from SSF1, seeks each region of all image volumes for a local path that is consistent with the subset of measured data. Once the vessel’s motion path is identified, the data are discretized into a series of datasets based on when the corresponding projection rays were measured. Each volume dataset in the series undergoes the process of spatial deformation by the motion field. This allows the motion state to be mapped from the respective time to the central reference time that is determined by the prescribed cardiac phase^21,30^. Therefore, SSF2 can not only further reduce coronary artery artifacts due to motion in patients with high heart rates but it also improves the image quality of other cardiac vascular structures such as valves and cardiac muscles on cardiovascular CT images. Our results support earlier findings^22^ that, compared with SSF1, SSF2 further improved the image quality of not only coronary arteries but also of all valves and other cardiac structures.

As the heart rate increased, the median ERD became significantly shorter on SSF2- than standard reconstruction images. A shorter ERD results in sharper edges^33,34,37^. Therefore, the ERD quantitatively demonstrates that SSF2 reconstruction reduced motion artifacts attributable to a high heart rate. The concept of quantifying motion artifacts via the EDR is further supported by the significant difference (*p* < 0.001) in our qualitative evaluation data.

We found that the dispersion of the area and perimeter of the aortic annulus measured by two observers was significantly smaller on SSF2- than standard reconstruction images regardless of the heart rate. This suggests that SSF2 reconstruction facilitates measurement of the aortic annulus by reducing motion artifacts. Although we did not assess its measurement accuracy by comparing our findings with the reference standard for transesophageal echocardiography^32^, we expect that the routine use of SSF2 will result in higher measurement accuracy than conventional standard reconstruction imaging in patients with severe aortic stenosis.

Although cardiac CT is the reference standard for the workup of TAVI candidates scheduled for an investigation of the aortic root^1,2^, motion artifacts reduce the accuracy of aortic annulus sizing and directly impact on patient outcome after TAVI procedure^2,7–9^. As a result of evaluating the dispersion between the two reconstructions with respect to the sizing of the aortic annulus, SSF2 was significantly smaller than standard regardless of the patients’ heart rate or R-R interval. For TAVI planning, we still tend to use systolic imaging for the measurements^2,4,30,31^ and the aortic annulus seems to be better delineated when SSF2 is used. Therefore, SSF2 may contribute to improving the accuracy of sizing of the aortic annulus.

During catheter-based implantation especially of the balloon-expandable prosthesis, it is important to use a fluoroscopic projection that provides an exact orthogonal view onto the aortic annular plane^2,8,9^. Because CT offers a 3D dataset, it allows identification of appropriate projection angles that will provide an orthogonal view onto the aortic valve plane^38,39^. Others^1,2^ reported that appropriate angles can be predicted from pre-TAVI cardiac CT scans when dedicated automated software programs are used. However, this is only possible if the aortic valve plane is accurately defined on the CT scan^39,40^. As SSF2 accurately delineated the aortic annulus, its application may result in a more accurate automatic processing of CT images for TAVI.

As renal dysfunction is relatively common in elderly patients scheduled for TAVI, a low-contrast protocol is recommended^41^. SSF2 reconstruction may be appropriate in TAVI candidates with renal dysfunction because it not only improves the image quality but also reduces the need for rescanning.

To avoid the potential impact of SSF2 reconstruction on quantitative measurements of the ERD, we measured the CT number in the ascending aorta, the image noise, and the CNR on SSF2 reconstructed images. We found that CNR was equivalent between scans subjected to standard- or SSF2 reconstruction irrespective of the patients’ heart rate, confirming that SSF2 corrected only the motion artifacts and that it did not affect other parameters.

Our study has some limitations. First, our study population was relatively small and our investigation was based on single-center experiences. Second, because we had no reference standard such as transesophageal echocardiographs for all patients, our ability to determine whether the subjectively judged aortic annulus image quality improves the accuracy of measurements. Third, we only evaluated the differences between two reconstruction algorithms applied on the same CT scanner and we did not compare our findings with those made on, for example, dual-source CT scans. Finally, additional studies are underway to determine whether the robustness of SSF2 reconstruction allows lowering the preset padding range prior to scanning, thereby minimizing the required radiation dose.

## Conclusions

In conclusion, our findings suggest that the SSF2 algorithm was superior to standard reconstruction because it improved the image quality and reduces motion artifacts especially in patients with a high heart rate or a 40% R-R interval. These findings may help SSF2 improve the accuracy of sizing of the aortic annulus prior to TAVI (“Supplementary Information”).

## Supplementary Information

Supplementary Information.

## Data Availability

All relevant data are within the main manuscript.

## Abbreviations

bpm: Beats per minute
CT: Computed tomography
CNR: Contrast-to-noise ratio
ERD: Edge rise distance
MPR: Multiplanar reconstruction
ROI: Region of interest
SD: Standard deviation
SSF: SnapShot Freeze
TAVI: Transcatheter aortic valve implantation
